# A longitudinal study of plasma BAFF levels in mothers and their infants in Uganda, and correlations with subsets of B cells

**DOI:** 10.1371/journal.pone.0245431

**Published:** 2021-01-19

**Authors:** Caroline Rönnberg, Allan Lugaajju, Anna Nyman, Ulf Hammar, Matteo Bottai, Maximilian Julius Lautenbach, Christopher Sundling, Fred Kironde, Kristina E. M. Persson

**Affiliations:** 1 Department of Microbiology, Tumor, and Cell Biology, Karolinska Institutet, Stockholm, Sweden; 2 Division of Infectious Diseases, Department of Medicine Solna, and Center for Molecular Medicine, Karolinska Institutet, Stockholm, Sweden; 3 Makerere University, Kampala, Uganda; 4 Department of Laboratory Medicine, Lund University, Skåne University Hospital, Lund, Sweden; 5 Division of Biostatistics, Institute of Environmental Medicine, Karolinska Institutet, Stockholm, Sweden; 6 Department of Infectious Diseases, Karolinska University Hospital, Stockholm, Sweden; 7 Habib Medical School, Islamic University in Uganda (IUIU), Mbale, Uganda; Institut Cochin, FRANCE

## Abstract

Malaria is a potentially life-threatening disease with approximately half of the world’s population at risk. Young children and pregnant women are hit hardest by the disease. B cells and antibodies are part of an adaptive immune response protecting individuals continuously exposed to the parasite. An infection with *Plasmodium falciparum* can cause dysregulation of B cell homeostasis, while antibodies are known to be key in controlling symptoms and parasitemia. BAFF is an instrumental cytokine for the development and maintenance of B cells. Pregnancy alters the immune status and renders previously clinically immune women at risk of severe malaria, potentially due to altered B cell responses associated with changes in BAFF levels. In this prospective study, we investigated the levels of BAFF in a malaria-endemic area in mothers and their infants from birth up to 9 months. We found that BAFF-levels are significantly higher in infants than in mothers. BAFF is highest in cord blood and then drops rapidly, but remains significantly higher in infants compared to mothers even at 9 months of age. We further correlated BAFF levels to *P*. *falciparum-*specific antibody levels and B cell frequencies and found a negative correlation between BAFF and both *P*. *falciparum*-specific and total proportions of IgG^+^ memory B cells, as well as CD27^−^ memory B cells, indicating that exposure to both malaria and other diseases affect the development of B-cell memory and that BAFF plays a part in this. In conclusion, we have provided new information on how natural immunity against malaria is formed.

## Introduction

*Plasmodium falciparum* malaria remains a major global health concern and is estimated to cause over 400 000 deaths every year [1]. Children under five and pregnant women in sub-Saharan Africa are most severely affected by the disease. Malaria during pregnancy can cause symptoms of disease even in women who grew up in malaria-endemic areas and thus acquired clinical immunity prior to the pregnancy [2]. The placenta offers a new breeding ground for the malaria parasite with resulting erythrocytic sequestration through pregnancy-specific virulence factors, such as placental adhesion by the VAR2CSA protein [3]. The consequences of placental malaria include fetal death, preterm delivery and low birth weight of the infant.

Humoral immunity is a key component in naturally acquired immunity to clinical malaria. This has been shown by passive transfer of immunoglobulins from malaria-immune adults to children with acute malaria, resulting in a drop in parasite levels and clinical improvement [4]. Also, in primigravidae women, the risk of complications in the fetus as well as in the mother is higher than in multigravidae women, and antibodies against VAR2CSA have been shown to correlate with protection [5–8]. Achieving clinical immunity to malaria takes years of exposure, and antibody responses are known to be short-lived in the absence of continuous infections, especially in children, even though more long-lived responses have also been seen [9–12]. B cells, as the source of antibodies, have been shown to be dysregulated by malaria infection [13–15]. The mechanisms behind, and consequences of this disrupted B cell homeostasis are currently unclear.

B cell activating factor (BAFF) is a cytokine belonging to the tumor necrosis factor (TNF) family of ligands, and is released by myeloid cells such as monocytes, macrophages and dendritic cells [16]. BAFF is known as a survival factor for B cells and is effective throughout the developmental stages of a B cell after release from the bone marrow [17, 18]. BAFF maintains B cell homeostasis, supports the survival of plasma cells [19], and promotes class switch recombination [20]. Both membrane-bound and soluble forms of BAFF are functionally active, either directly by cross-linking one of three different receptors on the B cells via surface-expressed BAFF, or indirectly by enzymatic release of soluble BAFF [21].

BAFF and the related cytokine APRIL (a proliferation-inducing ligand) are both TNF family cytokines with important roles in promoting peripheral B cell survival, development, and activation. BAFF exerts its impact on B cells by binding to one of the following three receptors: the BAFF-R, transmembrane activator and CAML interactor (TACI), and B cell maturation Ag (BCMA), whereas APRIL binds TACI and BCMA. In mice, BCMA is thought to function mainly as a plasma cell-specific survival receptor [22, 23]. In humans, plasma cells and memory B cells express both TACI and BCMA, while BAFF-R is expressed on most mature B cells beyond pre-B cells, except for plasma cells [24, 25]. In line with this, BAFF-R deletion causes the loss of peripheral B cells beyond the transitional stage [26]. BAFF-R is thus crucial for the survival and development of mature naive B cells, but it is not needed for the maintenance of memory B cells and long-lived plasma cells in the bone marrow [27]. In contrast to systemic BAFF, which is essential for homeostatic control of pre-immune B cell pools, locally produced BAFF is involved in regulating aspects of humoral immune responses [22]. Mesenchymal cells in term placental villi have been shown to be major sites of BAFF and APRIL synthesis [28].

During the acute phase of malaria, patients have been shown to have high levels of BAFF in plasma, and children have also been shown to have elevated expression levels of TACI and BCMA while BAFF-R was decreased [29]. This finding was corroborated using controlled human malaria infection [30]. In contrast, malaria in mice resulted in a decrease in the proportion of dendritic cells that expressed BAFF, leading to a reduced ability of these dendritic cells to support memory B cell differentiation into antibody secreting cells [31]. Increased expression of BAFF mRNA in placental samples from women with placental malaria has also been reported [32].

Previous studies investigating the levels of BAFF in cord blood from children born in non-endemic areas have shown increased levels of BAFF compared to adults [33–35]. However, the opposite has also been reported [36]. The levels of BAFF in newborns from malaria-endemic areas is of interest given the potential *in utero* exposure to an infection skewing the B cell response. A recently published study in Kenya showed that levels of BAFF in cord blood of healthy newborns were double that of their mothers’ levels at birth [37]. In the current study, we sought to measure the plasma BAFF-levels in mothers and their infants in a malaria-endemic setting. Variations in BAFF-levels were compared over time during the first nine months of the babies´ lives. Furthermore, levels of BAFF were correlated to *P*. *falciparum-*specific B cell phenotypes and outlined in relation to data on antibody titers to *P*. *falciparum* schizont extract [38, 39].

## Materials and methods

### Study design

Mothers and their infants were enrolled between March 2012 and July 2013 for a longitudinal study at Kasangati Health Centre, Uganda. Kasangati is located about 20 km from Kampala and receives patients from a surrounding peri-urban area with mesoendemic malaria. Peak transmission occurs after the rainy seasons in February-March and September-October. The study cohort is described in detail elsewhere [39]. In short, 131 mother-infant pairs were enrolled at birth and followed up at three subsequent time points coinciding with the regular vaccination schedule. Infants were sampled at birth (cord blood) and then at 10 weeks, 6 months and 9 months of age. Their mothers were sampled at delivery and 9 months after delivery. None of the study individuals had any signs of severe infection at any given point of sampling. Peripheral blood mononuclear cells (PBMC), and at the first sampling of infants, cord blood mononuclear cells (CBMC) and plasma were separated and stored. Rapid diagnostic tests (RDT) for malaria were used for all study participants on all sampling occasions. Positive RDT results were verified by light microscopy. A written informed consent was obtained from all the mothers prior to study enrollment. Out of the 131 samples, we included 109 with adequate volumes to perform measurements of BAFF at all time points. The study was approved by the School of Medicine Research and Ethics Committee (SOMREC) of Makerere University, the Uganda National Council of Science and Technology (approval 2011–114) and by Regionala Etikprövningsnämnden in Stockholm, Sweden 2014/478-32.

### BAFF ELISA

Plasma samples were tested for the concentration of BAFF (Quantikine ELISA kit, R&D Systems) according to the manufacturer’s instructions with the following modification: the volume of standard was doubled while retaining the same concentration. Plasma samples were diluted 1:5 for the majority of samples and 1:10 in case of plasma shortage. Optical density was determined at 450 nm using a SPECTRA max340PC384 microplate reader. BAFF concentrations were calculated using SoftMax pro software. All samples were run in either duplicate or triplicate.

### *P*. *falciparum* schizont extract ELISA

Total anti-*P*. *falciparum* IgG and IgM in blood plasma were measured as described [39]. Briefly, microtiter plate wells were coated with schizont extract and blocked with 5% skimmed milk (Sigma) for IgG and super block dry blend (Thermo Scientific) for IgM. Diluted plasma samples were added to the wells in the microtiter plates and incubated at room temperature for 1 hour. Plates were washed 4 times between the incubation steps and subsequently incubated with peroxidase-conjugated goat anti-human IgG/IgM (Sigma) and rewashed. Bound secondary antibody was quantified using TMB (3,3′,5,5′-Tetramethylbenzidine) substrate (Promega). Optical density (OD) was read at 450 nm. All samples were analyzed twice and the means of the OD used in the analysis.

### B cell phenotyping

PBMC were phenotyped using a LSRII flow cytometer (Becton-Dickinson Immuno Cytometry Systems, San Jose, USA) as described [39]. The following fluorochrome-conjugated monoclonal antibodies were used for B cell phenotyping: CD19 PE-CF594-clone HIB19, CD20 V450-clone L27, CD27 PE-Cy7-clone M-T271, and IgG FITC-clone G18-145 (all from BD). B cells specific for *P*. *falciparum* were identified utilizing Quantum dots (Invitrogen) conjugated to extract of schizont- and trophozoite-stage parasites as described in detail previously [38]. Proportions of B lymphocytes (defined as CD19^+^ cells) specific for *P*. *falciparum* (Pf+) were defined in the following cell compartments: IgG positive memory B cells (IgG^+^ MBC) (CD19^+^CD20^+^CD27^+^IgG^+^), IgG negative memory B cells (non-IgG^+^ MBC) (CD19^+^CD20^+^CD27^+^IgG^−^), naïve B cells (CD19^+^CD20^+^CD27^−^IgG^−^), plasma cells/blasts (CD19^+^CD20^−^CD27^+^IgG^−^), and CD27^−^ MBC, including atypical memory B cells (CD19^+^CD20^+^CD27^−^IgG^+^). Data was processed using FLOWJO software (Tree Star Inc, San Carlos, CA, USA).

### Detection of malaria parasites

Parasite prevalence in the cohort was determined using pLDH/HRP2 RDT strips (Combo Rapid Diagnostic Test of Premier Medical Corporation Limited, India) for all individuals on every sampling occasion. For verification and determining parasitemia, Giemsa-stained thick and thin blood smears were prepared from all RDT-positive individuals.

### Statistical analysis

Measures were taken on mothers at birth (M0), infants at birth (B0), infants at 10 weeks (B2.5), infants at 6 months (B6), infants at 9 months (B9) and mothers at 9 months (M9). We report the mean and range as descriptive summary measures of numeric variables. In a first analysis, we used a linear random-intercept model to estimate mean BAFF concentration across different time-points (birth, 10 weeks, 6 months and 9 months), between mothers and infants, and between parasitemic and non-parasitemic individuals. The random intercept was included to take the potential intra-individual dependence into account. BAFF concentration was the dependent variable. The independent variables were the indicator variables for the four time points, an indicator for whether a measure was taken on the mother or the infant, an indicator whether the individual was parasitemic or not, and interaction terms between the mother indicator and the time-point indicators. The birth time point was the reference category.

In a second analysis we used linear random-intercept models to estimate mean proportions of the following variables: IgG^+^ MBC, non-IgG^+^ MBC, CD27^−^ MBC, naïve B cells and plasma cells/blasts. We evaluated the change in mean over time. Correlations between mean proportion of cell compartments and BAFF concentration were assessed using rho = and obtained p-values were adjusted for multiple testing using the Benjamini-Hochberg [40] correction. BAFF concentration was also correlated to levels of anti-schizont-IgM and IgG. BAFF levels were prior log_10_ transformed to reduce the impact of outliers. All statistical analyses were performed using Stata 13, Graphpad Prism and R [41].

## Results

### Concentration of BAFF in mothers and infants

**Fig 1:**
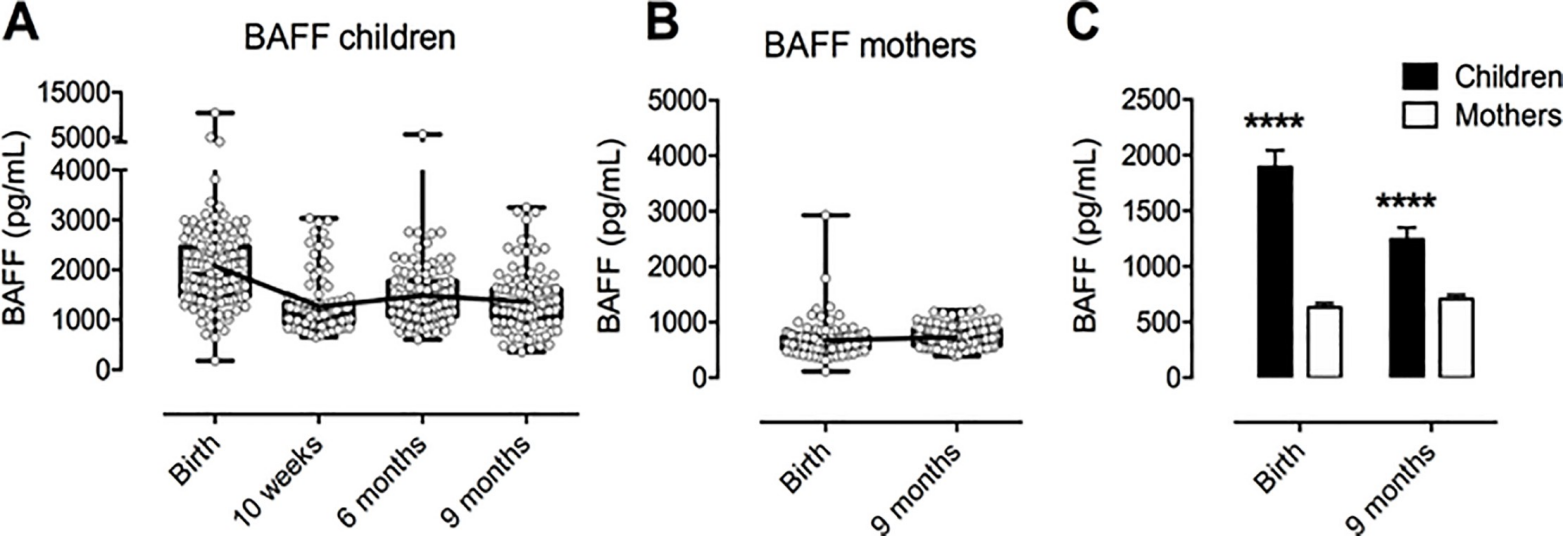
Distribution of individual and mean levels of measured BAFF concentrations for children and mothers at each time point. A) BAFF levels in children over the course of the study. B) BAFF levels in mothers at birth and 9 months postpartum. C) Comparison of mean BAFF levels in children and mothers at birth and at 9 months using unpaired t test with Welch’s correction. **** p<0.0001. All figs show bars and errors with geometric mean + 95% confidence interval. For A, B: Mean values indicated by interconnecting line.

BAFF levels were measured in the plasma from 109 mother-infant pairs. Individual levels of BAFF in the infants varied between 182 pg/mL and 10472 pg/mL, with both the highest and lowest values measured at birth (Fig 1A). In mothers, the lowest and highest levels of BAFF measured were 113 pg/mL and 2928 pg/mL, respectively (Fig 1B). Both values were measured at birth. The mean concentration of BAFF for all infants and time points were as follows: Birth: 2075 pg/mL, 10 weeks: 1265 pg/mL, 6 months: 1483 pg/mL and 9 months: 1363 pg/mL. Mean BAFF-concentrations for mothers were at Birth: 670 pg/mL and at 9 months: 733 pg/mL. Mean BAFF-levels in children were significantly higher than in mothers at birth with a difference of 1405 pg/mL (p<0.0001; 95% confidence interval 1590 to 1219) (Fig 1C). The difference between mean BAFF-levels was smaller at 9 months; 631 pg/mL (95% confidence interval 747 to 515) but remained significantly higher (p<0.0001).

Mean BAFF concentration in infants was reduced by 810 pg/mL from baseline to 10 weeks (p<0.001; 95% CI: 623 to 998), and levelled off from that point onward. Conversely, no significant change between birth and nine months was detected in mothers (p = 0.38).

### Parasitemia effect on mean BAFF concentration

During the entire study period, 17 individuals had a positive RDT out of which 14 were found to have parasites in the blood by microscopy. Six mothers and one infant were parasitemic at birth. Parasites were found in one infant at 10 weeks, three infants at 6 months and one infant and two mothers at 9 months. Since the samples containing parasites were very few, we analysed these values of BAFF together as a group and compared to those samples that did not contain parasites. No significant difference in BAFF concentration was found between parasitemic and non-parasitemic samples (p = 0.11).

### Correlation between *P*. *falciparum* schizont-specific antibodies and BAFF levels

Antibody levels to schizont extract in all individuals at each time point were previously reported [39]. Here, we statistically reanalyzed the data and correlated with BAFF levels to investigate potential associations. Mean schizont-specific IgG levels in infants were highest at birth. These levels decreased at 10 weeks with a subsequent tendency towards an increase at 9 months of age (S1 Fig). Mean IgM levels in infants were lowest at birth and then steadily increased over the time of the study period. In mothers, mean levels of both IgG and IgM were higher than in infants and stable over both time points. Hence, from the ELISA data it is clear that the infants were exposed to malaria during the first 9 months of life, however levels of antibodies did not reach adult levels during the study period (S1 Fig). The correlation analysis for BAFF and antibodies to schizont extract generated no associations between BAFF levels and IgG (S1 and S2 Tables), while, there was a significant negative association between BAFF levels and IgM at birth in infants and a positive correlation at 9 months in mothers (S3 and S4 Tables).

### Correlation between total and *P*. *falciparum*-positive B cell subsets and BAFF levels

**Fig 2:**
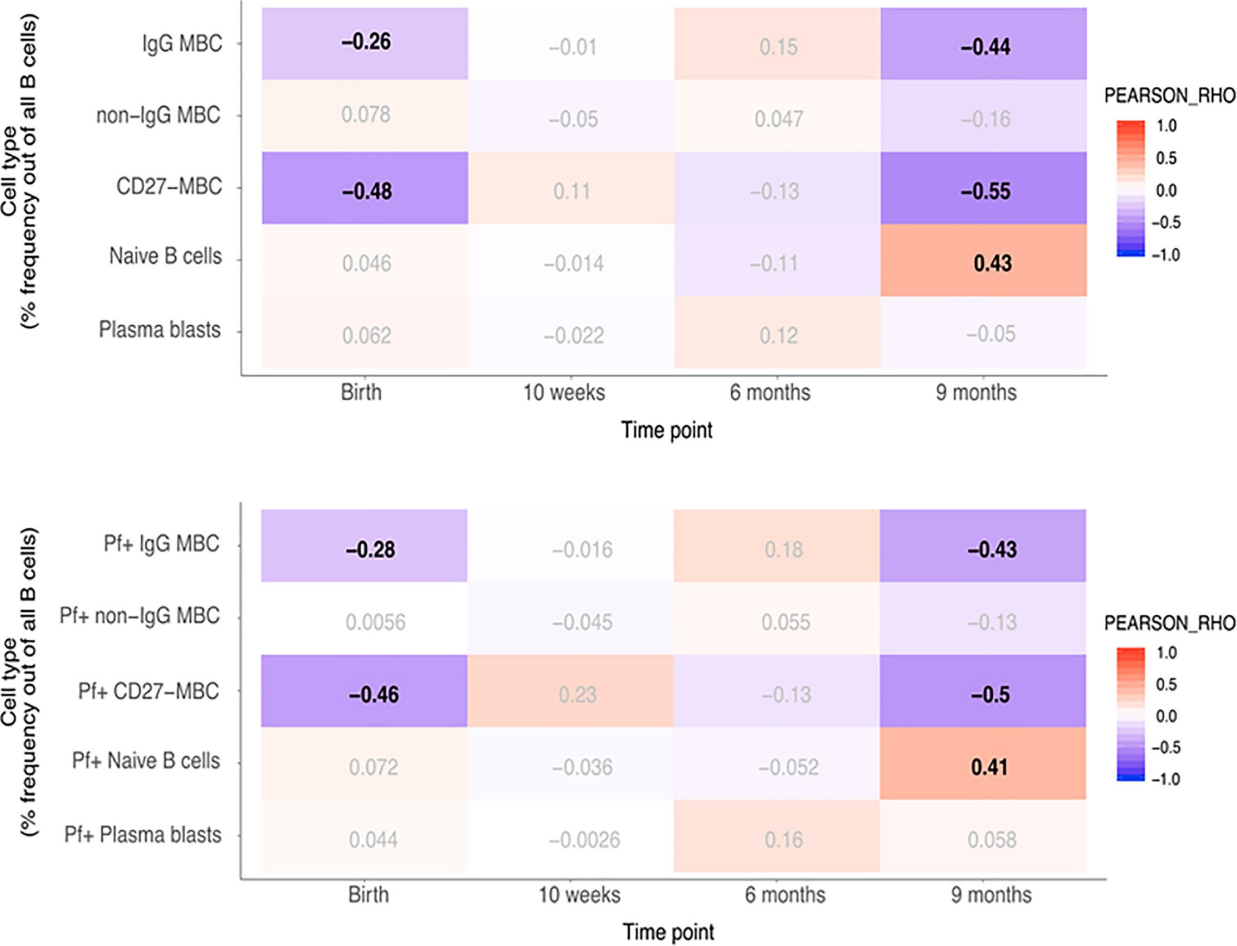
Correlation heatmap of BAFF concentration and given subsets of B cells in infants. A. Pearson correlation with adjusted p-value for multiple testing using the Benjamini-Hochberg method. Pearson’ rho values with a false discovery rate (FDR) < 0.05 in bold. B. Correlation heatmap of BAFF concentration and Pf+ proportions of given subsets of B cells in infants. Pearson correlation with adjusted p-value for multiple testing using the Benjamini-Hochberg method. Pearson’ rho values with a false discovery rate (FDR) < 0.05 in bold.

**Fig 3:**
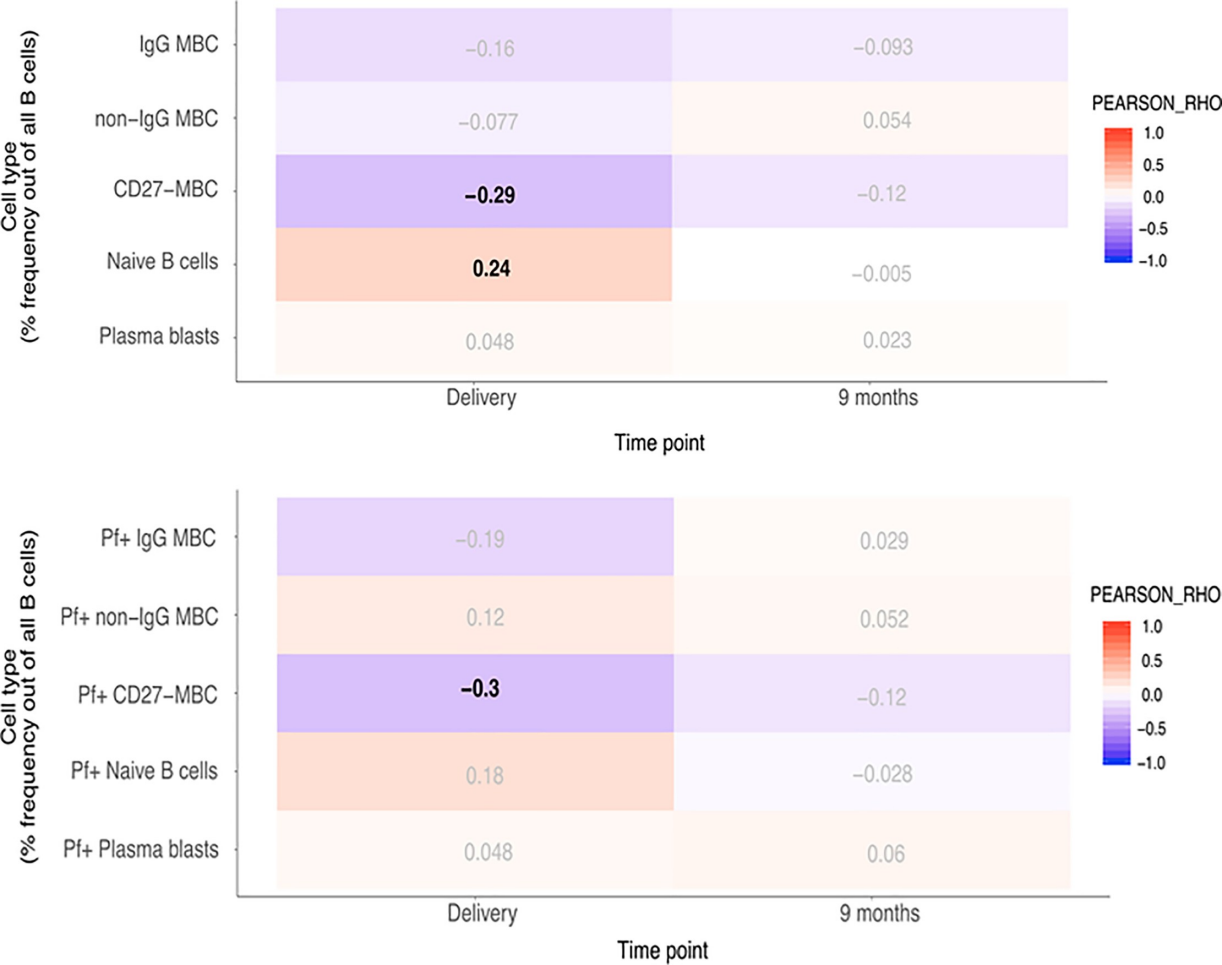
Correlation heatmap of BAFF concentration and given subsets of B cells in mothers. A. Pearson correlation with adjusted p-value for multiple testing using the Benjamini-Hochberg method. Pearson’ rho values with a false discovery rate (FDR) < 0.05 in bold. B. Correlation heatmap of BAFF concentration and Pf+ proportions of given subsets of B cells in mothers. Pearson correlation with adjusted p-value for multiple testing using the Benjamini-Hochberg method. Pearson’ rho values with a false discovery rate (FDR) < 0.05 in bold.

As BAFF has a role in B cell survival and differentiation, we correlated BAFF levels with total and Pf+ B cell subsets, previously reported for these individuals [39]. Pearson correlation analyses were performed with p-value correction for multiple testing (FDR < 0.05) and we considered rho -values between 0.3–0.7 associations of moderate strength. Mean proportions of IgG^+^ MBC, non-IgG^+^ MBC, Naïve B cells, CD27^−^ MBC and plasma cells within the CD19+ B cell compartment in infants are shown in S2 Fig, and the gating strategy used is shown in S3 Fig. At birth, changes in BAFF concentrations in newborns were negatively correlated with CD27^−^ MBC (rho = –0.48) including Pf+ proportions (rho = –0.46). In children at nine months, changes in BAFF concentrations were negatively correlated with IgG^+^ MBC and CD27^−^ MBC (rho = –0.44 and rho = –0.55, respectively) including Pf+ proportions for both these compartments (rho = –0.43 and rho = –0.5, respectively) (Fig 2). The reported associations were of moderate strength and highly significant. All statistical results are outlined in S6–S9 Tables. In addition, we found a significant, albeit weak negative association at birth in the IgG^+^ MBC subset of both total and Pf+ cells (rho = –0.26 and rho = –0.28, respectively). Conversely, in children at nine months, a positive correlation with BAFF-levels was found in the naïve B cell subset in both total and Pf+ compartments (rho = 0.43 and rho = 0.41, respectively). In mothers at delivery, changes in BAFF concentration were weakly to moderately negatively correlated with CD27^−^ MBC (rho = –0.29), and Pf+ CD27^−^ MBC (rho = –0.30) (Fig 3). On the other hand, naïve B cells were weakly positively correlated with BAFF-levels at delivery (rho = 0.24).

## Discussion

In the current study, we analyzed blood plasma samples of mothers and their infants which were prospectively collected at four time points from birth up to 9 months. Study individuals were consecutively recruited throughout the year in an area of mesoendemic malaria transmission in Uganda. The follow-up samples were therefore collected at various periods of the year following the study schedule regardless of endemicity. Mean levels of BAFF for each time point are thus the result of individual levels at varying exposure to malaria.

Previous studies investigating BAFF concentrations in infants and mothers have been conducted both in non-endemic countries in Europe [33–35], and in Kenya [37]. Consistent with these publications, our results show overall higher levels of BAFF in infants than in mothers, possibly mirroring a developing immune system. In our relatively large cohort of newborns and their mothers, mean BAFF-levels in cord blood were 2075 pg/mL and in mothers 670 pg/mL. These results are in the same order as those found both in malaria-free areas and in Kenya [34, 35, 37]. Mesenchymal cells in term placental villi are major sites of BAFF synthesis [28] which may explain the substantially higher levels of BAFF seen in newborns compared to their mothers. Levels of BAFF were highest in cord blood and decreased when measured in peripheral blood at 10 weeks, subsequently levelling off at 6 to 9 months. This finding is also in line with a longitudinal study in Sweden [35]. Interestingly, the lowest individual concentrations of BAFF measured among either infants or mothers was at birth, and was in the same mother-infant pair, potentially indicating a strong genetic or environmental component in determining BAFF levels. The highest individual levels of BAFF were also measured at birth but did not, however, correspond within the mother-infant pairs. Yeo et al. showed that cord blood of healthy Kenyan neonates had higher levels of BAFF when compared with United States neonates born to healthy mothers [37]. Also, cord blood from Kenyan neonates exposed to HIV and/or CMV and/or *P*.*falciparum* in utero had higher levels of BAFF than US cord blood, while there was no significant difference between US cord blood and Kenyan cord blood from neonates exposed to *P*.*falciparum* only, although the small sample size may not suffice to show any potential difference. This suggests that malaria exposure among other factors, could be a driving factor causing higher levels of BAFF in cord blood of infants born in endemic areas. Other infections, such as helminths, must be taken into consideration as these are very common in tropical areas and may elicit a considerable immune response in addition to malaria.

Nduati et al. showed that, during the acute phase of malaria infection, children had high plasma BAFF levels and elevated TACI and BCMA expression levels, while the BAFF-R expression level decreased [29]. Also, monocytes exposed to the schizont fraction of malaria-infected erythrocytes *in vitro* strongly expressed BAFF and induced B-cell proliferation and IgG secretion [42]. In our study, although we did have a few individuals with detectable parasitemia, we did not observe significantly increased BAFF-levels in these individuals compared to non-parasitemic individuals (S5 Table). This is not surprising however due to several reasons. Firstly, none of the individuals had overt signs of infection on the scheduled day for sampling and so the ones with positive RDT and/or microscopy likely represent a carrier state rather than acute infection. This finding is interesting in itself, as it indicates that the mere presence of parasites does not induce increased levels of BAFF, if not accompanied by clinical symptoms of disease. Secondly, very few individuals within the cohort were found positive for malaria and were spread out over the study period, which restricts comparative analysis. In addition, pregnant women at Kasangati Health Center are offered intermittent preventive treatment (IPT) in order to prevent clinical malaria during pregnancy. Although we had no data on compliance with IPT within our cohort, experience indicates that IPT is generally well established and practiced in this area. This could then explain the low number of malaria positive mothers at birth. Bearing in mind the small number of parasitemic individuals, it is still possible that acute febrile malaria results in increased plasma BAFF-levels.

Pregnancy in malaria-naive women has been associated with a significant expansion of switched MBC and a decrease of naïve B cells, while malaria-exposed pregnant women had more atypical MBC and fewer marginal zone–like MBC [43]. In our cohort, both IgM and IgG antibody levels to schizont extract were stable in the mothers, likely indicating repeated previous exposure to malaria. In infants, IgG which crosses the placenta, was high in cord blood and then decreased as expected. IgM, however, which is produced by the child and thus the most appropriate indicator of previous malaria exposure, showed a low level at birth and then increased towards the 9-month sampling. This dynamic can probably be attributed to the loss of maternal protection and formation of the child’s own malaria-specific immune response. B cell data in infants initially showed high proportions of naïve B cells and low proportions of IgG^+^ MBC, non-IgG^+^ MBC and CD27^−^ MBC in cord blood. While the frequency of naive B cells steadily decreased over time, IgG^+^ MBC and CD27^−^ MBC increased. These shifts in B cell proportions appear plausible as a reflection of the developing humoral response with gradual exposure to pathogens. Non-IgG^+^ MBC rapidly increased from birth to 10 weeks of age and then decreased over the following 9 months. Plasma cells, on the other hand, showed a marginal increase from cord blood levels at the two middle sampling points and then dropped again at 9 months. Plasma cell numbers are however, normally very low in peripheral blood and usually only expand in connection with a recent, or ongoing, infection [44]. The rapid increase followed by a slow decrease of non-IgG^+^ MBC may mirror the clonal expansion of B cells responding to unknown antigens and subsequent isotype switching. Non-IgG^+^ MBC could also be innate-like B cells that expand rapidly with subsequent levelling of the proportions as other more specific subsets expand.

In infants, significant negative correlations were found between BAFF levels and both total and Pf+ IgG^+^ MBC and CD27^−^ MBC at 9 months. The high BAFF levels could indicate a more immature immune system where a larger proportion of B cells are still naïve, while continuous pathogen exposure will drive maturation of the immune response toward more memory cells and activated B cells, and likely simultaneously reducing available peripheral BAFF levels. Based on previous findings [15], the CD27^−^ MBC subset in our cohort can be expected to be enriched for atypical MBC, which are known to be functionally impaired compared to classical MBC [45, 46]. Considering the nature of malaria exposure and the consistent correlation between BAFF and both total and Pf+ proportions of IgG^+^ MBC and CD27^−^ MBC, one can hypothesize that BAFF in relation to *P*. *falciparum* could have a specific impact on the B cell response. Conversely, there was a significant positive correlation between BAFF levels and naïve B cells at 9 months, including the Pf+ proportion. This association could potentially be explained by higher levels of BAFF leading to an increase in naïve B cells, or that these individuals had a more immature immune response, retaining more of the early elevated BAFF levels. There was a weak albeit significant negative correlation between BAFF levels and IgM antibodies to schizont extract in cord blood which could indicate that high levels of BAFF is important for mounting an immune response against malaria. In mothers, weak but significant negative correlations were found between BAFF-levels and CD27^−^ MBC at delivery, but not at 9 months. This might be due to the absence of parasitemia following IPT leading to lower BAFF-levels. IgM antibodies to schizont extract were only weakly associated with BAFF-levels at 9 months which might represent a return to a normal immune state post pregnancy. In controlled human malaria infection there was a positive correlation between increased plasma BAFF and IFN-γ levels, and atypical B cells which also showed the strongest proliferative response of all memory B cell subsets upon infection [30]. These experimental malaria-infections are conducted on healthy, malaria-naïve adults, however, and cannot be directly transferred to interpret immune responses in endemic areas. Nevertheless, in natural *P*. *falciparum* infection we have previously shown that CD11c^+^ B cells enriched for atypical B cells are the most proliferative during acute malaria [47]. However, the interplay between BAFF and immune cells in adults may be different from that occurring in infants and older children.

As reviewed by Sakai et al. [18], BAFF levels in serum have been shown to increase during viral infections, such as HIV and HCV. Similarly, bacterial infections such as *M*. *pneumoniae* and *M*. *tuberculosis* can cause increased levels of BAFF in respiratory tract samples, and cerebrospinal fluid from patients with neuroborreliosis show increased levels of BAFF. A number of autoimmune disorders display dysregulation of BAFF, and overexpression of BAFF has been implicated in the pathogenesis of systemic lupus erythematosus (SLE) [48]. In HIV, hypergammaglobulinemia appears to be a result of a dysregulated B cell compartment characterized by the BAFF-induced expansion of marginal zone B cells [49]. *P*. *falciparum* infection is also accompanied by hypergammaglobulinemia, and it has been shown to be a consequence of parasite-induced polyclonal B cell activation [14, 50]. Analogous to an infection with HIV, the non-IgG^+^ MBC population seen in our study may constitute a similar mechanism of marginal zone B cells expanding in response to BAFF. Our results suggest both stimulatory and regulatory functions of BAFF in infants and in the aftermath of pregnancy, although from this study we cannot establish a causal link between BAFF and different subpopulations of B cells.

A limitation of this study is the restricted selection of markers used to characterize the B cell populations. In the absence of a wider repertoire of B cell markers, some of the cells that are classified as naïve B cells may indeed belong to a pool of more immature B cells [51]. However, it was shown before that proportions of naïve B cells decrease with age during childhood [52], overlapping with what we also observe here. Similarly, as mentioned previously, the non-IgG^+^ memory B-cells may represent innate-like/MZ-like B cells. Taken together, we believe that the results obtained in our study indicate several interesting potential associations that can constitute the basis for further studies, especially since our results include both *P*. *falciparum*-positive and total proportions of B cells. Ideally though, future studies could include more B cell markers such as CD24/CD38, CD21 and IgD for better separation of subpopulations of B cells.

In conclusion, plasma BAFF levels in infants from malaria-endemic areas are significantly higher than their mothers’ BAFF levels, both at birth and after 9 months. BAFF levels correlate with IgG^+^ MBC and CD27^−^ MBC of both total and Pf+ compartments in infants, suggesting that BAFF could play a role in the development of naturally acquired immunity against malaria. Additional prospective studies in older children in endemic and non-endemic areas, including immunophenotyping and monitoring of BAFF, in conjunction with active and passive malaria detection are warranted to further establish the role of BAFF in malaria.

## Supporting information

S1 Fig Mean *P*. *falciparum* schizont-specific antibody levels in mothers and children. The levels of antibodies in the mothers were stable over time, while antibodies in the children changed as can be expected due to having maternal IgG at birth, and then being exposed to malaria early in life.
(PDF)

S2 Fig Mean proportions of given B cell compartments in mothers and children. (PDF)

S3 Fig Gating strategy used for B cell phenotyping. (PDF)

S1 Table Correlation between BAFF-levels and schizont-specific IgG-levels in infants. (DOCX)

S2 Table Correlation between BAFF-levels and schizont-specific IgG-levels in mothers. (DOCX)

S3 Table Correlation between BAFF-levels and schizont-specific IgM-levels in infants. Boxes with significant correlations are filled with light grey.
(DOCX)

S4 Table Correlation between BAFF-levels and schizont-specific IgM-levels in mothers. Boxes with significant correlations are filled with light grey.
(DOCX)

S5 Table Parasitemia (number of infected RBC/μL of blood) for individual infants and mothers and their corresponding levels of BAFF. (DOCX)

S6 Table Correlation between BAFF-levels and subsets of B cells in infants. Boxes with significant correlations are filled with light grey.
(DOCX)

S7 Table Correlation between BAFF-levels and Pf+ subsets of B cells in infants. Boxes with significant correlations are filled with light grey.
(DOCX)

S8 Table Correlation between BAFF-levels and subsets of B cells in mothers. Boxes with significant correlations are filled with light grey.
(DOCX)

S9 Table Correlation between BAFF-levels and subsets of Pf+ B cells in mothers. Boxes with significant correlations are filled with light grey.
(DOCX)

S1 File (PDF)
