# Supplementary material for: A longitudinal study of plasma BAFF levels in mothers and their infants in Uganda, and correlations with subsets of B cells
Source: PLoS One. 2021 Jan 19;16(1):e0245431. doi: 10.1371/journal.pone.0245431 (PMC7815132; doi:10.1371/journal.pone.0245431)
Supplement: S1 Fig — The levels of antibodies in the mothers were stable over time, while antibodies in the children changed as can be expected due to having maternal IgG at birth, and then being exposed to malaria early in life. (PDF) [file pone.0245431.s001.pdf]

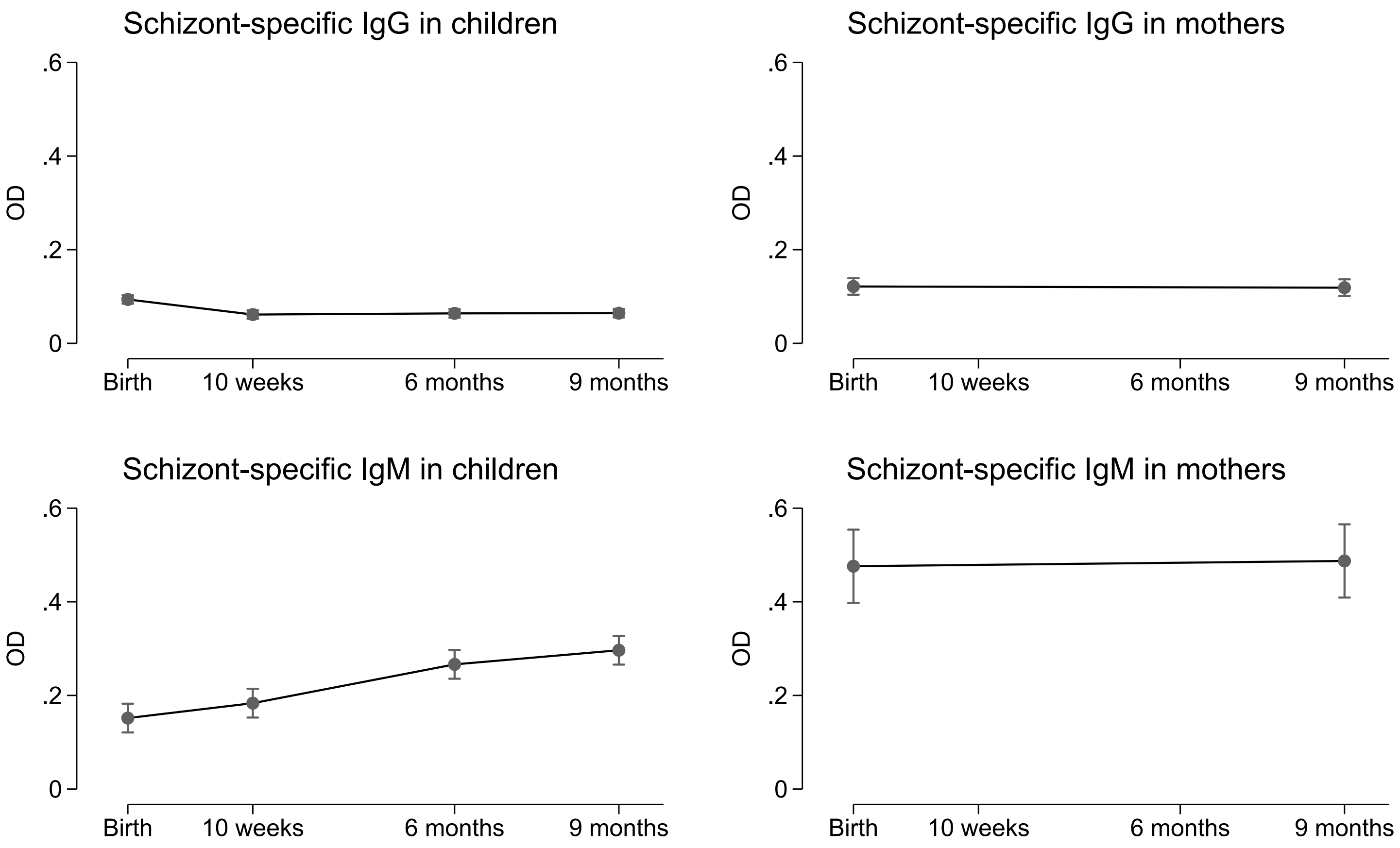
Schizont-specific IgG in children
Schizont-specific IgG in mothers
Schizont-specific IgM in children
Schizont-specific IgM in mothers
OD
.6
.4
.2
0
Birth
10 weeks
6 months
9 months
