# Supplementary material for: A longitudinal study of plasma BAFF levels in mothers and their infants in Uganda, and correlations with subsets of B cells
Source: PLoS One. 2021 Jan 19;16(1):e0245431. doi: 10.1371/journal.pone.0245431 (PMC7815132; doi:10.1371/journal.pone.0245431)
Supplement: S2 Fig — (PDF) [file pone.0245431.s002.pdf]

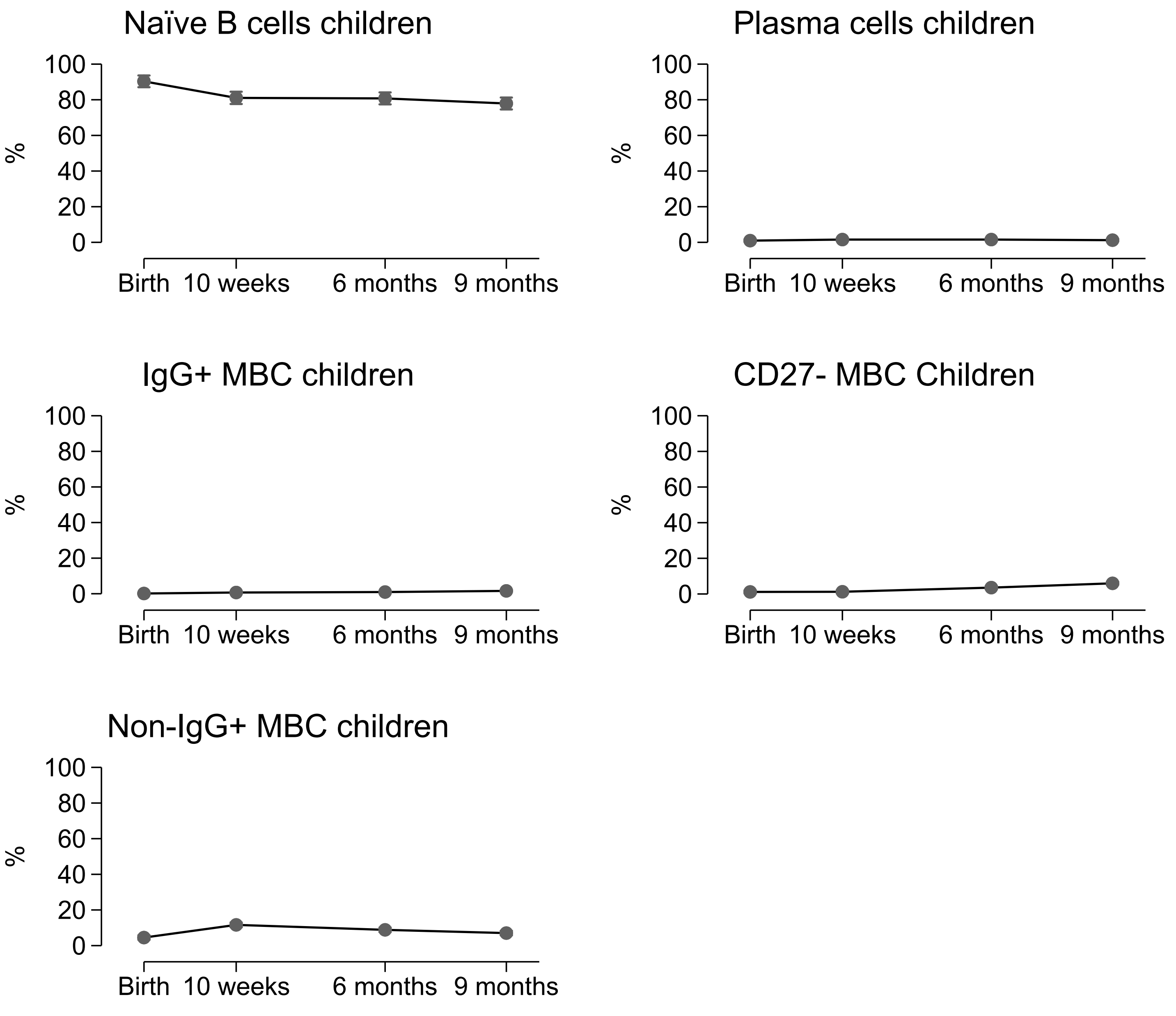

Naïve B cells children
Plasma cells children
IgG+ MBC children
CD27- MBC Children
Non-IgG+ MBC children
%
100
80
60
40
20
0
Birth
10 weeks
6 months
9 months
