# Supplementary figures and images for: A longitudinal study of plasma BAFF levels in mothers and their infants in Uganda, and correlations with subsets of B cells
Source: PLoS One. 2021 Jan 19;16(1):e0245431. doi: 10.1371/journal.pone.0245431 (PMC7815132; doi:10.1371/journal.pone.0245431)

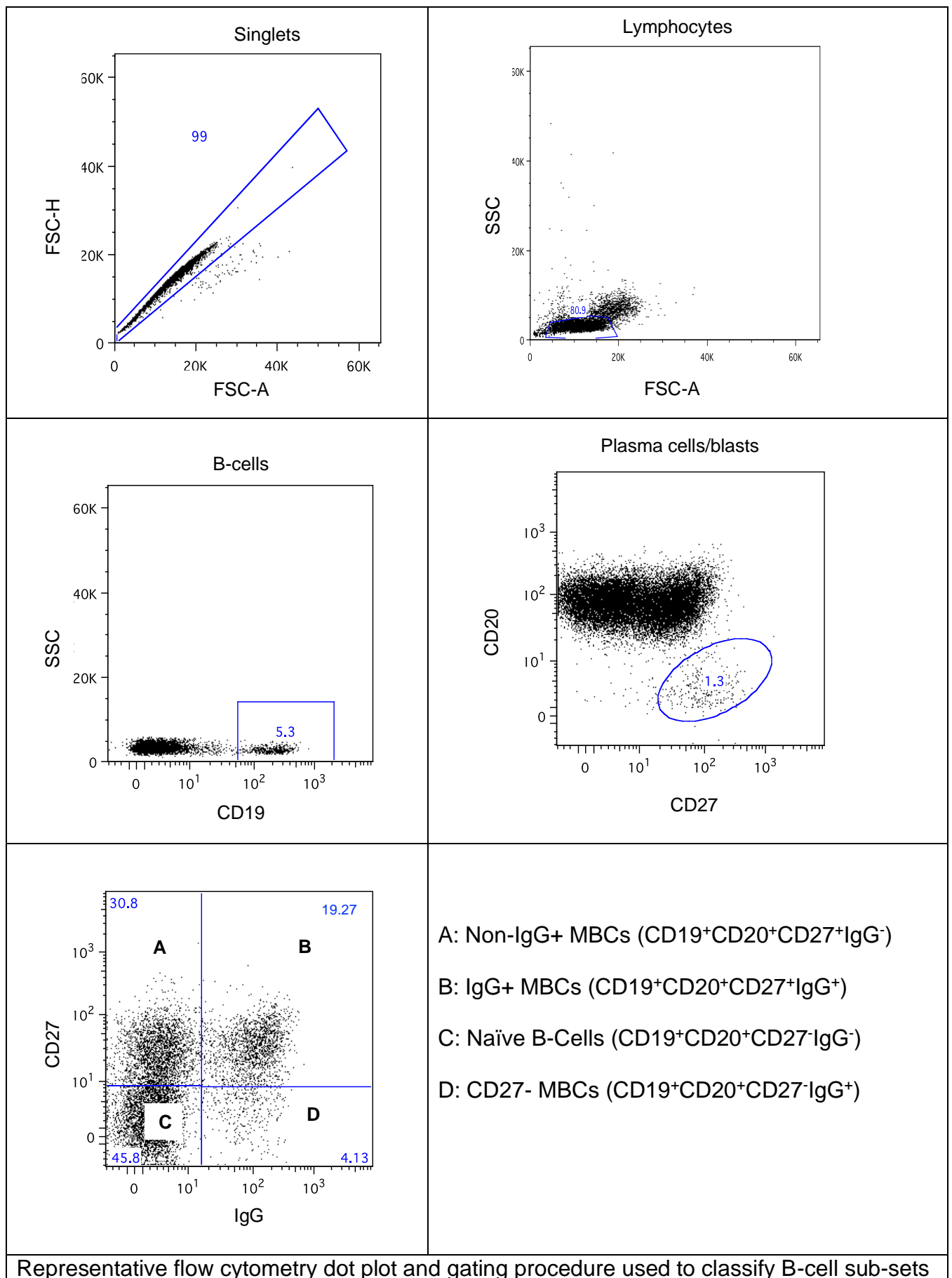

Representative flow cytometry dot plot and gating procedure used to classify B-cell sub-sets

Supplement: S3 Fig — (PDF) [file pone.0245431.s003.pdf]
