# Supplementary material for: A longitudinal study of plasma BAFF levels in mothers and their infants in Uganda, and correlations with subsets of B cells
Source: PLoS One. 2021 Jan 19;16(1):e0245431. doi: 10.1371/journal.pone.0245431 (PMC7815132; doi:10.1371/journal.pone.0245431)
Supplement: S1 Table — (DOCX) [file pone.0245431.s004.docx]

**S1 Table. Correlation between BAFF-levels and schizont-specific IgG-levels in infants.**

|  | **Pearson(r)** |
| --- | --- |
| **Cord blood**  **BAFF vs IgG** | 0.04  p=0.68 |
| **10 weeks**  **BAFF vs IgG** | 0.02  p=0.87 |
| **6 months**  **BAFF vs IgG** | 0.14  p=0.18 |
| **9 months**  **BAFF vs IgG** | 0.11  p=0.28 |
