# Supplementary material for: A longitudinal study of plasma BAFF levels in mothers and their infants in Uganda, and correlations with subsets of B cells
Source: PLoS One. 2021 Jan 19;16(1):e0245431. doi: 10.1371/journal.pone.0245431 (PMC7815132; doi:10.1371/journal.pone.0245431)
Supplement: S2 Table — (DOCX) [file pone.0245431.s005.docx]

**S2 Table. Correlation between BAFF-levels and schizont-specific IgG-levels in mothers.**

|  | **Pearson(r)** |
| --- | --- |
| **Delivery**  **BAFF vs IgG** | -0.04  p=0.71 |
| **9 months after delivery**  **BAFF vs IgG** | 0.17  p=0.10 |
|  |  |
