# Supplementary material for: A longitudinal study of plasma BAFF levels in mothers and their infants in Uganda, and correlations with subsets of B cells
Source: PLoS One. 2021 Jan 19;16(1):e0245431. doi: 10.1371/journal.pone.0245431 (PMC7815132; doi:10.1371/journal.pone.0245431)
Supplement: S3 Table — Boxes with significant correlations are filled with light grey. (DOCX) [file pone.0245431.s006.docx]

**S3 Table. Correlation between BAFF-levels and schizont-specific IgM-levels in infants.** Boxes with significant correlations are filled with light grey.

|  | **Pearson(r)** |
| --- | --- |
| **Cord blood**  **BAFF vs IgM** | -0.34  p=0.0003 |
| **10 weeks**  **BAFF vs IgM** | -0.17  p=0.08 |
| **6 months**  **BAFF vs IgM** | -0.16  p=0.10 |
| **9 months**  **BAFF vs IgM** | -0.18  p=0.07 |
