# Supplementary material for: A longitudinal study of plasma BAFF levels in mothers and their infants in Uganda, and correlations with subsets of B cells
Source: PLoS One. 2021 Jan 19;16(1):e0245431. doi: 10.1371/journal.pone.0245431 (PMC7815132; doi:10.1371/journal.pone.0245431)
Supplement: S4 Table — Boxes with significant correlations are filled with light grey. (DOCX) [file pone.0245431.s007.docx]

**S4 Table. Correlation between BAFF-levels and schizont-specific IgM-levels in mothers.** Boxes with significant correlations are filled with light grey.

|  | **Pearson(r)** |
| --- | --- |
| **Delivery**  **BAFF vs IgM** | -0.02  p=0.82 |
| **9 months after delivery**  **BAFF vs IgM** | 0.31  p=0.001 |
