# Supplementary material for: A longitudinal study of plasma BAFF levels in mothers and their infants in Uganda, and correlations with subsets of B cells
Source: PLoS One. 2021 Jan 19;16(1):e0245431. doi: 10.1371/journal.pone.0245431 (PMC7815132; doi:10.1371/journal.pone.0245431)
Supplement: S5 Table — (DOCX) [file pone.0245431.s008.docx]

**S5 Table. Parasitemia (number of infected RBC/µL of blood) for individual infants and mothers and their corresponding levels of BAFF.**

| **Time Course** | **Sample** **ID** | **Parasitemia** | **BAFF level (pg/mL)** |
| --- | --- | --- | --- |
| Cord blood | KB 101 | 13000 | 883 |
| Infants at 10 weeks | KB 120 | 9560 | 2737 |
| Infants at 6 months | KB 56 | 91760 | 5674 |
|  | KB 118 | 22624 | 1244 |
|  | KB 125 | 3840 | 806 |
| Infants at 9 months | KB 57 | 5080 | 1172 |
| Mothers at delivery | KM 12 | 48 | 633 |
|  | KM 32 | 16 | 499 |
|  | KM 35 | 240 | 763 |
|  | KM 41 | 64 | 805 |
|  | KM 58 | 32 | 611 |
|  | KM 60 | 2760 | 1099 |
| Mothers 9 months post partum | KM 91 | 1520 | 673 |
|  | KM 96 | 800 | 732 |
