# Supplementary material for: A longitudinal study of plasma BAFF levels in mothers and their infants in Uganda, and correlations with subsets of B cells
Source: PLoS One. 2021 Jan 19;16(1):e0245431. doi: 10.1371/journal.pone.0245431 (PMC7815132; doi:10.1371/journal.pone.0245431)
Supplement: S6 Table — Boxes with significant correlations are filled with light grey. (DOCX) [file pone.0245431.s009.docx]

**S6 Table: Correlation between BAFF-levels and subsets of B cells in infants**. Boxes with significant correlations are filled with light grey.

|  | **Time** | **Protein** | **Celltype** | **PEARSON_RHO** | **P-VALUE** | **FDR** | **FDR_sci** | **RHO_sci** |
| --- | --- | --- | --- | --- | --- | --- | --- | --- |
| 1 | Birth | BAFF | CD27- MBC | -0.48 | 2.46e-07 | 1.48e-06 | 1.5e-06 | -4.8e-01 |
| 2 | Birth | BAFF | IgG MBC | -0.26 | 0.01 | 0.03 | 2.7e-02 | -2.6e-01 |
| 3 | Birth | BAFF | non-IgG MBC | 0.08 | 0.43 | 0.77 | 7.7e-01 | 7.8e-02 |
| 4 | Birth | BAFF | Plasma cells/blasts | 0.06 | 0.54 | 0.77 | 7.7e-01 | 6.2e-02 |
| 5 | Birth | BAFF | Naive B cells | 0.05 | 0.64 | 0.77 | 7.7e-01 | 4.6e-02 |
| 6 | 10 weeks | BAFF | CD27- MBC | 0.11 | 0.31 | 0.93 | 9.3e-01 | 1.1e-01 |
| 7 | 10 weeks | BAFF | non-IgG MBC | -0.05 | 0.65 | 0.93 | 9.3e-01 | -5.0e-02 |
| 8 | 10 weeks | BAFF | Plasma cells/blasts | -0.02 | 0.84 | 0.93 | 9.3e-01 | -2.2e-02 |
| 9 | 10 weeks | BAFF | Naive B cells | -0.01 | 0.90 | 0.93 | 9.3e-01 | -1.4e-02 |
| 10 | 10 weeks | BAFF | IgG MBC | -0.01 | 0.93 | 0.93 | 9.3e-01 | -1.0e-02 |
| 11 | 6 months | BAFF | IgG MBC | 0.15 | 0.14 | 0.32 | 3.2e-01 | 1.5e-01 |
| 12 | 6 months | BAFF | CD27- MBC | -0.13 | 0.21 | 0.32 | 3.2e-01 | -1.3e-01 |
| 13 | 6 months | BAFF | Plasma cells/blasts | 0.12 | 0.23 | 0.32 | 3.2e-01 | 1.2e-01 |
| 14 | 6 months | BAFF | Naive B cells | -0.11 | 0.27 | 0.32 | 3.2e-01 | -1.1e-01 |
| 15 | 6 months | BAFF | non-IgG MBC | 0.05 | 0.65 | 0.65 | 6.5e-01 | 4.7e-02 |
| 16 | 9 months | BAFF | CD27- MBC | -0.55 | 5.75e-09 | 3.45e-08 | 3.4e-08 | -5.5e-01 |
| 17 | 9 months | BAFF | IgG MBC | -0.44 | 8.22e-06 | 2.47e-05 | 2.5e-05 | -4.4e-01 |
| 18 | 9 months | BAFF | Naive B cells | 0.43 | 1.33e-05 | 2.65e-05 | 2.7e-05 | 4.3e-01 |
| 19 | 9 months | BAFF | non-IgG MBC | -0.16 | 0.12 | 0.14 | 1.4e-01 | -1.6e-01 |
| 20 | 9 months | BAFF | Plasma cells/blasts | -0.05 | 0.63 | 0.63 | 6.3e-01 | -5.0e-02 |
