# Supplementary material for: A longitudinal study of plasma BAFF levels in mothers and their infants in Uganda, and correlations with subsets of B cells
Source: PLoS One. 2021 Jan 19;16(1):e0245431. doi: 10.1371/journal.pone.0245431 (PMC7815132; doi:10.1371/journal.pone.0245431)
Supplement: S7 Table — Boxes with significant correlations are filled with light grey. (DOCX) [file pone.0245431.s010.docx]

**S7 Table:** **Correlation between BAFF-levels and Pf+ subsets of B cells in infants.** Boxes with significant correlations are filled with light grey.

|  | **Time** | **Protein** | **Celltype** | **PEARSON_RHO** | **P-VALUE** | **FDR** | **FDR_sci** | **RHO_sci** |
| --- | --- | --- | --- | --- | --- | --- | --- | --- |
| 1 | Birth | BAFF | Pf+ CD27- MBC | -0.46 | 1.08e-06 | 6.51e-06 | 6.5e-06 | -4.6e-01 |
| 2 | Birth | BAFF | Pf+ IgG MBC | -0.28 | < 0.01 | 0.01 | 1.2e-02 | -2.8e-01 |
| 3 | Birth | BAFF | Pf+ Naive B cells | 0.07 | 0.47 | 0.71 | 7.1e-01 | 7.2e-02 |
| 4 | Birth | BAFF | Pf+ Plasma cells/blasts | 0.04 | 0.66 | 0.79 | 7.9e-01 | 4.4e-02 |
| 5 | Birth | BAFF | Pf+ non-IgG MBC | 0.01 | 0.96 | 0.96 | 9.6e-01 | 5.6e-03 |
| 6 | 10 weeks | BAFF | Pf+ CD27- MBC | 0.23 | 0.04 | 0.23 | 2.3e-01 | 2.3e-01 |
| 7 | 10 weeks | BAFF | Pf+ non-IgG MBC | -0.05 | 0.68 | 0.98 | 9.8e-01 | -4.5e-02 |
| 8 | 10 weeks | BAFF | Pf+ Naive B cells | -0.04 | 0.75 | 0.98 | 9.8e-01 | -3.6e-02 |
| 9 | 10 weeks | BAFF | Pf+ IgG MBC | -0.02 | 0.89 | 0.98 | 9.8e-01 | -1.6e-02 |
| 10 | 10 weeks | BAFF | Pf+ Plasma cells/blasts | < -0.01 | 0.98 | 0.98 | 9.8e-01 | -2.6e-03 |
| 11 | 6 months | BAFF | Pf+ IgG MBC | 0.18 | 0.07 | 0.36 | 3.6e-01 | 1.8e-01 |
| 12 | 6 months | BAFF | Pf+ Plasma cells/blasts | 0.16 | 0.12 | 0.36 | 3.6e-01 | 1.6e-01 |
| 13 | 6 months | BAFF | Pf+ CD27- MBC | -0.13 | 0.20 | 0.40 | 4.0e-01 | -1.3e-01 |
| 14 | 6 months | BAFF | Pf+ non-IgG MBC | 0.06 | 0.59 | 0.73 | 7.3e-01 | 5.5e-02 |
| 15 | 6 months | BAFF | Pf+ Naive B cells | -0.05 | 0.61 | 0.73 | 7.3e-01 | -5.2e-02 |
| 16 | 9 months | BAFF | Pf+ CD27- MBC | -0.50 | 2.32e-07 | 1.39e-06 | 1.4e-06 | -5.0e-01 |
| 17 | 9 months | BAFF | Pf+ IgG MBC | -0.43 | 1.26e-05 | 3.78e-05 | 3.8e-05 | -4.3e-01 |
| 18 | 9 months | BAFF | Pf+ Naive B cells | 0.41 | 4.30e-05 | 8.60e-05 | 8.6e-05 | 4.1e-01 |
| 19 | 9 months | BAFF | Pf+ non-IgG MBC | -0.13 | 0.20 | 0.30 | 3.0e-01 | -1.3e-01 |
| 20 | 9 months | BAFF | Pf+ Plasma cells/blasts | 0.06 | 0.58 | 0.69 | 6.9e-01 | 5.8e-02 |
