# Supplementary material for: A longitudinal study of plasma BAFF levels in mothers and their infants in Uganda, and correlations with subsets of B cells
Source: PLoS One. 2021 Jan 19;16(1):e0245431. doi: 10.1371/journal.pone.0245431 (PMC7815132; doi:10.1371/journal.pone.0245431)
Supplement: S8 Table — Boxes with significant correlations are filled with light grey. (DOCX) [file pone.0245431.s011.docx]

|  | **Time** | **Protein** | **Celltype** | **PEARSON_RHO** | **P-VALUE** | **FDR** | **FDR_sci** | **RHO_sci** |
| --- | --- | --- | --- | --- | --- | --- | --- | --- |
| 1 | Delivery | BAFF | CD27- MBC | -0.29 | < 0.01 | 0.01 | 1.5e-02 | -2.9e-01 |
| 2 | Delivery | BAFF | Naive B cells | 0.24 | 0.02 | 0.05 | 4.8e-02 | 2.4e-01 |
| 3 | Delivery | BAFF | IgG MBC | -0.16 | 0.10 | 0.21 | 2.1e-01 | -1.6e-01 |
| 4 | Delivery | BAFF | non-IgG MBC | -0.08 | 0.43 | 0.52 | 5.2e-01 | -7.7e-02 |
| 5 | Delivery | BAFF | Plasma cells/blasts | 0.05 | 0.63 | 0.63 | 6.3e-01 | 4.8e-02 |
| 6 | 9 months | BAFF | CD27- MBC | -0.12 | 0.26 | 0.76 | 7.6e-01 | -1.2e-01 |
| 7 | 9 months | BAFF | IgG MBC | -0.09 | 0.38 | 0.76 | 7.6e-01 | -9.3e-02 |
| 8 | 9 months | BAFF | non-IgG MBC | 0.05 | 0.61 | 0.92 | 9.2e-01 | 5.4e-02 |
| 9 | 9 months | BAFF | Plasma cells/blasts | 0.02 | 0.83 | 0.96 | 9.6e-01 | 2.3e-02 |
| 10 | 9 months | BAFF | Naive B cells | -0.01 | 0.96 | 0.96 | 9.6e-01 | -5.0e-03 |

**S8 Table: Correlation between BAFF-levels and subsets of B cells in mothers**. Boxes with significant correlations are filled with light grey.
