# Supplementary material for: A longitudinal study of plasma BAFF levels in mothers and their infants in Uganda, and correlations with subsets of B cells
Source: PLoS One. 2021 Jan 19;16(1):e0245431. doi: 10.1371/journal.pone.0245431 (PMC7815132; doi:10.1371/journal.pone.0245431)
Supplement: S9 Table — Boxes with significant correlations are filled with light grey. (DOCX) [file pone.0245431.s012.docx]

|  | **Time** | **Protein** | **Celltype** | **PEARSON_RHO** | **P-VALUE** | **FDR** | **FDR_sci** | **RHO_sci** |
| --- | --- | --- | --- | --- | --- | --- | --- | --- |
| 1 | Delivery | BAFF | Pf+ CD27- MBC | -0.30 | < 0.01 | 0.01 | 1.4e-02 | -3.0e-01 |
| 2 | Delivery | BAFF | Pf+ IgG MBC | -0.19 | 0.05 | 0.10 | 9.6e-02 | -1.9e-01 |
| 3 | Delivery | BAFF | Pf+ Naive B cells | 0.18 | 0.06 | 0.10 | 9.6e-02 | 1.8e-01 |
| 4 | Delivery | BAFF | Pf+ non-IgG MBC | 0.12 | 0.23 | 0.28 | 2.8e-01 | 1.2e-01 |
| 5 | Delivery | BAFF | Pf+ Plasma cells/blasts | 0.05 | 0.63 | 0.63 | 6.3e-01 | 4.8e-02 |
| 6 | 9 months | BAFF | Pf+ CD27- MBC | -0.12 | 0.24 | 0.71 | 7.1e-01 | -1.2e-01 |
| 7 | 9 months | BAFF | Pf+ Plasma cells/blasts | 0.06 | 0.56 | 0.79 | 7.9e-01 | 6.0e-02 |
| 8 | 9 months | BAFF | Pf+ non-IgG MBC | 0.05 | 0.62 | 0.79 | 7.9e-01 | 5.2e-02 |
| 9 | 9 months | BAFF | Pf+ IgG MBC | 0.03 | 0.78 | 0.79 | 7.9e-01 | 2.9e-02 |
| 10 | 9 months | BAFF | Pf+ Naive B cells | -0.03 | 0.79 | 0.79 | 7.9e-01 | -2.8e-02 |

**S9 Table:** **Correlation between BAFF-levels and subsets of Pf+ B cells in mothers**. Boxes with significant correlations are filled with light grey.
