# Supplementary material for: A longitudinal study of plasma BAFF levels in mothers and their infants in Uganda, and correlations with subsets of B cells
Source: PLoS One. 2021 Jan 19;16(1):e0245431. doi: 10.1371/journal.pone.0245431 (PMC7815132; doi:10.1371/journal.pone.0245431)
Supplement: S1 File — (PDF) [file pone.0245431.s013.pdf]

Supplementary Data file 1: BAFF levels (pg/mL)

BabyNr	Cord blood	Baby10week	Baby6month	Baby9month	*MotherNr*	MotherDeliv	Mother9month
KB001	182	867	1166	951	KM001	113	657
KB002	2140				KM002	661	
KB003				2359	KM003	560	557
KB004	1454	878	1060	1188	KM004	540	1006
KB005	1337	1019	762	2042	KM005	530	1002
KB006	1430	1226	1214	1097	KM006	564	649
KB007	1674				KM007	690	
KB008	2642	1478	894	1569	KM008	665	678
KB009	1452	865	1094	1136	KM009	686	949
KB010	1397	892	1358	1150	KM010	717	957
KB011	3064	1963	1431	2060	KM011	777	528
KB012	1906	1128	1173	1642	KM012	633	877
KB013	4075	1025	2252	1808	KM013	905	870
KB014	2447				KM014	647	
KB015	3120	1433	1137	876	KM015	547	1042
KB016	945	656		966	KM016	663	712
KB017	2250				KM017	496	
KB018	1487	1243	1277	1122	KM018	742	1158
KB019	1754	836	1317	1157	KM019	613	1061
KB020	2260	950	1132	912	KM020	645	592
KB021	1514	987	1210	937	KM021	715	928
KB022	5050			1311	KM022	619	746
KB023	1800	1033	977	1721	KM023	373	821
KB024	1015	947	895	1295	KM024	580	830
KB025	2123				KM025	873	
KB026	10472	1131	899	1791	KM026	895	601
KB027	2225	1322	1226	2591	KM027	613	753
KB028	1094				KM028	541	
KB029	1317				KM029	1002	
KB030	2316	1260	1606	1575	KM030	750	1003
KB031	791	1229	849		KM031	1125	856
KB032	1803	1040	2440	1178	KM032	499	984
KB033	2019	695	1780	1257	KM033	822	742
KB034	2586	1886	1901	1175	KM034	573	938
KB035	1871				KM035	763	
KB036	2141	1098	742	1315	KM036	665	912
KB037	2923	865		1052	KM037	772	849
KB038	2988	1329	1228	1192	KM038	660	711
KB039	2548	1023	1803		KM039	766	1220
KB040	1797	1038	1994	1313	KM040	727	616
KB041	2489	1161	1596	479	KM041	805	668
KB042	2597	1172	2222	1351	KM042	728	1134
KB043	2849	1256	2540	1364	KM043	735	924
KB044	1670				KM044	598	
KB045	2127		1629	1229	KM045	853	1191
KB046	1477				KM046	356	
KB047	2779		1729	1563	KM047	386	706
KB048	1519	1030	2232	1377	KM048	524	656
KB049	1356	1152	2224	1192	KM049	636	753
KB050	2204	977	1407	3007	KM050	560	861
KB051	2369	2466	1481	1614	KM051	690	1036
KB052	1217				KM052	457	
KB053	2442		1775	1446	KM053	587	785

KB054	1899				KM054	849	
KB055	2461	743	2224	1539	KM055	537	902
KB056	2670	1217	5674	1343	KM056	662	1167
KB057	1829	983	1464	1172	KM057	626	1181
KB058	2299	917	1793	1813	KM058	612	744
KB059	1733	1176	1216	1316	KM059	597	703
KB060	2179				KM060	1099	
KB061	1663				KM061	1232	
KB062	2981				KM062	531	
KB063	3098	1072	1913	3174	KM063	498	887
KB064	1410	1450	1598	2585	KM064	674	527
KB065	2845				KM065	471	
KB066	2220	1016	1075	3163	KM066	603	876
KB067	1589	840	2140	1400	KM067	731	569
KB068	1600	985	1713	1605	KM068	712	658
KB069	3268	1753	1955	1500	KM069	812	761
KB070	1786		1640	1172	KM070	736	828
KB071	2126	784	2079	773	KM071	492	835
KB072	2293	1179	1990	1145	KM072	684	536
KB073	2326	824	1011	2376	KM073	619	637
KB074	2963	904	1066	1893	KM074	1131	655
KB075	2295	839	1342	1087	KM075	740	392
KB076	2583	920	1417	1097	KM076	598	621
KB077	1104	707	1466	1431	KM077	707	845
KB078	2103	1353	1159	1620	KM078	656	806
KB079	2783	1076	1336	1915	KM079	524	750
KB080	1264		1560	1367	KM080	494	555
KB081	1097	1018	1662	1582	KM081	644	696
KB082	1648	907	1382	605	KM082	412	683
KB083	1880	952	1328	1169	KM083	599	735
KB084	1379	1019	1311	1984	KM084	580	716
KB085	2095		1185	1599	KM085	432	616
KB086	2087		1008	1162	KM086	591	598
KB087	1383	927	727	1048	KM087	622	487
KB088	1431	977	1349	3251	KM088	541	521
KB089	2083	944	829	2440	KM089	537	593
KB090	2073	1005	1126	2378	KM090	379	717
KB091	2001		1927	1547	KM091	537	673
KB092	1917	935	1245	1238	KM092	638	782
KB093	2323	1101	2727	414	KM093	594	521
KB094	1342	752	1334	869	KM094	564	526
KB095	1506	1348	1782	1647	KM095	501	633
KB096	2160	1307	894	2428	KM096	639	732
KB097	1780	945	1956	1397	KM097	523	584
KB098	3817	807	1578	1270	KM098	825	627
KB099	1891	984	777	425	KM099	489	543
KB100	2633	935	969	1116	KM100	396	456
KB101	883	1214	1413	1683	KM101	1790	456
KB102	1730	984	1490	477	KM102	901	751
KB103	1135	799	1545	754	KM103	313	647
KB104	712	937	762	1067	KM104	1270	490
KB105	1192	1509	2059	758	KM105	563	520
KB106	1376	1241	1477	824	KM106	544	418
KB107	2231	2052			KM107	505	
KB108	1805	864	2016	626	KM108	622	851
KB109	1438	2160	1039	947	KM109	436	586

KB110	1672	2982	1340	776	KM110	605	530
KB111	644	2310	859	643	KM111	2928	916
KB112	1643	1283	2760	962	KM112	751	559
KB113	1841	2548	1283	780	KM113	526	507
KB114	2492	2054	1655	638	KM114	702	454
KB115	863	1220	1629	1068	KM115	565	882
KB116	1553	1670	2756	1264	KM116	691	631
KB117	3358	3036	1133	1528	KM117	599	617
KB118	1611	1106	1244	504	KM118	778	739
KB119	1746	1191	1058	358	KM119	574	564
KB120	2795	2737	2748	583	KM120	603	487
KB121	2758	1232	857	679	KM121	727	620
KB122	2984	1701	1333	633	KM122	401	470
KB123	2713	1095	1150	1357	KM123	412	575
KB124	1586	1051	993	1296	KM124	690	551
KB125	1807	2956	806	1257	KM125	649	561
KB126	2873	2528	939	1173	KM126	1041	945
KB127	2364	2757	1735	1237	KM127	745	995
KB128	2444	1382	604	1175	KM128	708	839
KB129	1341	1370	843	1542	KM129	484	510
KB130	1291	1254	950	1940	KM130	526	588
KB131	2475	1385	1074	1756	KM131	854	812
